# New York City HIV Care Continuum Dashboards: Using Surveillance Data to Improve HIV Care Among People Living With HIV in New York City

**DOI:** 10.2196/13086

**Published:** 2019-06-19

**Authors:** Sarah L Braunstein, Karen Coeytaux, Charulata J Sabharwal, Qiang Xia, Rebekkah S Robbins, Beverly Obeng, Demetre C Daskalakis

**Affiliations:** 1 HIV Epidemiology and Field Services Program Bureau of HIV Prevention and Control New York City Department of Health and Mental Hygiene Long Island City, NY United States; 2 Rare Disease Registries Epidemiology and Biostatistics Group Global Medical Affairs Sanofi Genzyme Bridgewater, NJ United States; 3 Global Staph aureus Program Pfizer Vaccine Clinical Research & Development Pearl River, NY United States; 4 Division of Disease Control New York City Department of Health and Mental Hygiene Long Island City, NY United States

**Keywords:** HIV, surveillance, quality of care, best practices

## Abstract

**Background:**

HIV surveillance data can be used to improve patient outcomes.

**Objective:**

This study aimed to describe and present findings from the HIV care continuum dashboards (CCDs) initiative, which uses surveillance data to quantify and track outcomes for HIV patients at major clinical institutions in New York City.

**Methods:**

HIV surveillance data collected since 2011 were used to provide high-volume New York City clinical facilities with their performance on two key outcomes: linkage to care (LTC), among patients newly diagnosed with HIV and viral load suppression (VLS), among patients in HIV care.

**Results:**

The initiative included 21 facilities covering 33.78% (1135/3360) of new HIV diagnoses and 46.34% (28,405/61,298) of patients in HIV care in New York City in 2011 and was extended to a total of 47 sites covering 44.23% (1008/2279) of new diagnoses and 69.59% (43,897/63,083) of New York City patients in care in 2016. Since feedback of outcomes to providers began, aggregate LTC has improved by 1 percentage point and VLS by 16 percentage points.

**Conclusions:**

Disseminating information on key facility–level HIV outcomes promotes collaboration between public health and the clinical community to end the HIV epidemic. Similar initiatives can be adopted by other jurisdictions with mature surveillance systems and supportive laws and policies.

## Introduction

### Background

Over the last two decades, tremendous advances in HIV treatment have transformed HIV into a chronic, manageable condition [1]. Indeed, available HIV treatment and prevention programs allow persons living with HIV (PLWH) to live healthy lives and significantly reduce the spread of the disease [2-5]. Although HIV-related care outcomes have improved in recent years, HIV remains a significant public health challenge, with nearly 40,000 new infections in the United States in 2015 and over 6000 HIV-related deaths [6]. Ensuring that all PLWH receive high-quality medical care has become 1 of the top national priorities in bending the HIV prevalence curve and ending the epidemic [7].

The HIV care continuum has long been used as a framework for monitoring care-related outcomes for PLWH and informing the quality of HIV care delivered. It offers a comprehensive overview of the efficiency of HIV clinical management by highlighting stages between time of diagnosis and viral load suppression (VLS) that might need improvement. In particular, it provides an understanding of the performance on timely linkage to care (LTC), retention in care, and achievement of VLS [8,9]. In 2010, the National HIV/AIDS Strategy (NHAS) called for a more coordinated response to the HIV epidemic and set national goals to be reached by the end of 2015. NHAS was updated in 2015, and new targets to be reached by the end of 2020 were provided [10,11].

Many national and local strategies call for the use of HIV surveillance and other public health data to measure population-level progress toward goals and identify gaps along the HIV care continuum [12]. HIV surveillance data are widely available, population-based, and collected in a standardized way across jurisdictions; as such, they are uniquely suited for use in measuring care continuum outcomes at the local and national level. The HIV surveillance registry of the New York City Department of Health and Mental Hygiene (NYC DOHMH) represents a comprehensive and high-quality source of HIV data and includes detailed information on providers and clinical facilities associated with HIV diagnoses and HIV-related laboratory tests indicating HIV care. NYC DOHMH has used surveillance data extensively to guide its programmatic and field activities [13,14]. New York State public health law emphasizes (and allows) sharing of HIV surveillance data externally by the health department to enhance patient LTC and retention in care [15]. The HIV care continuum can be adapted to measure the effectiveness of individual clinical institutions, inform the quality of HIV care delivered by those institutions, and point to possible areas for improvement and intervention planning.

### Objectives

In 2012, NYC DOHMH launched the HIV care continuum dashboard (CCD) initiative, which consisted of providing selected New York City clinical providers with facility-level aggregate data reflecting the HIV care performance of their own facilities, their peers, and New York City as a whole. The goals of the CCD initiative were to monitor local progress toward the NHAS goals, to identify potential low-performing facilities in need of intervention, and to encourage adoption of best practices from high-performing facilities. We report on the implementation of this initiative by NYC DOHMH and the novel use of outcomes data to improve clinical management of HIV care by New York City providers.

## Methods

### Data Sources

New York State Public Health law requires named reporting to the NYC DOHMH of all HIV/AIDS diagnoses, all HIV-related illness, and all cluster of differentiation 4 (CD4), viral load (VL), and genotype tests conducted for New York City PLWH [15]. The NYC DOHMH manages the New York City HIV surveillance registry, which is continuously updated with demographic, clinical, and other information on persons receiving HIV care in New York City and meeting the HIV surveillance case definitions of the Centers for Disease Control and Prevention [16]. New York City Vital Statistics Registry and national death data (ie, National Death Index and Social Security Death Master File data) are routinely used to update death information on vital status in the registry. The New York City registry contains a cumulative total of more than 10 million HIV-related laboratory test results for over 240,000 individuals.

### Outcomes

The CCDs are facility-level performance reports derived from HIV surveillance data, displaying indicators on timely LTC, VLS, and VL below transmission threshold (BTT) for a specific 12-month period. In general, New York City HIV surveillance data are lagged to account for reporting delays as well as a standard dissemination timeline, such that data for the previous calendar year are released in December each year. The CCDs are released twice annually, in June and December. The June CCDs contain data for July to June of the previous year, and the December CCDs contain data for January to December of the previous year (eg, CCDs released in December 2012 contained data for January-December 2011, CCDs released in June 2013 contained data for July 2011-June 2012, and so on). CCDs are sent by email to each facility’s leadership, and VLS data from the December CCDs only are published on a dedicated page on the DOHMH website. [17]

**Figure 1:**
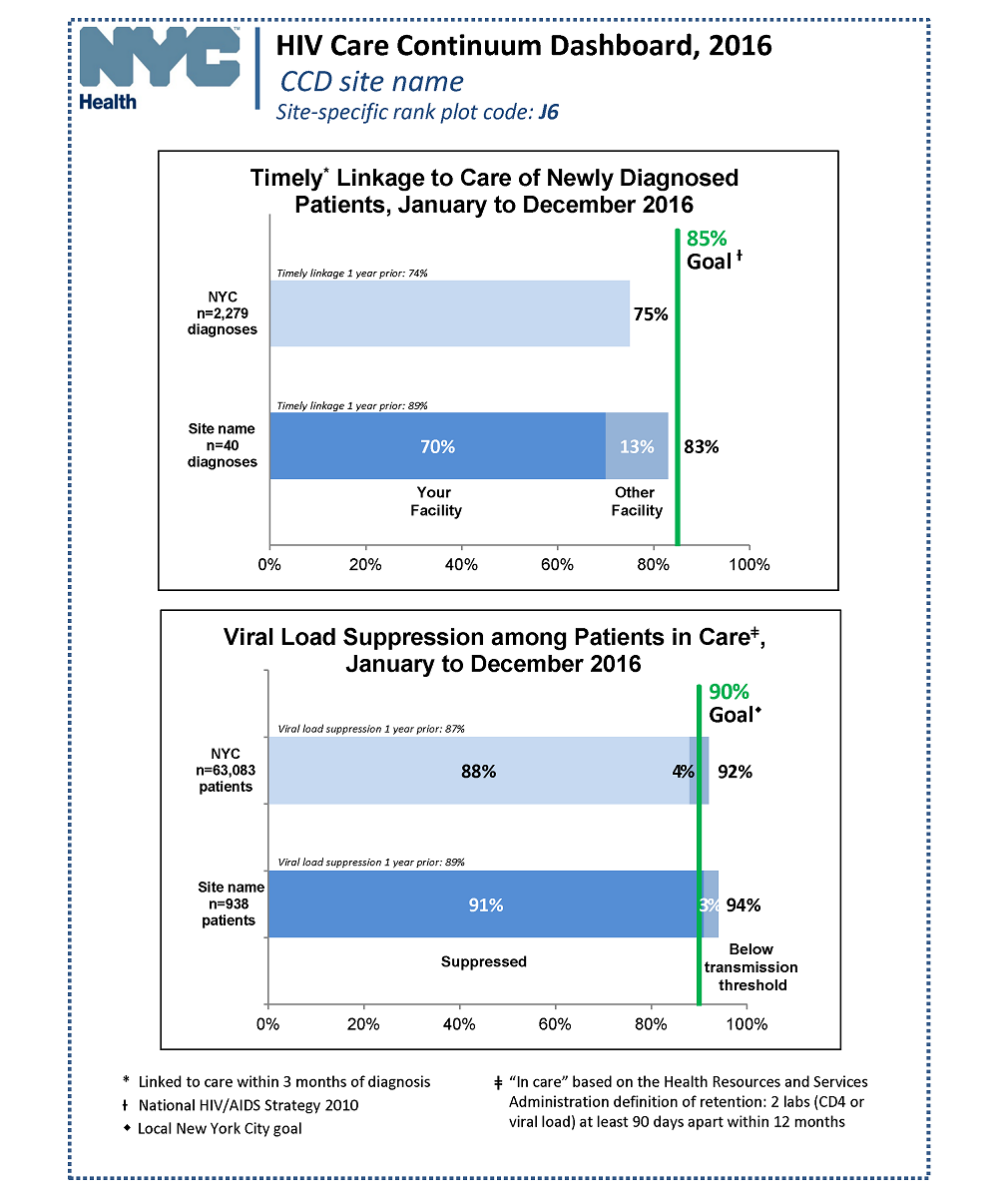
Overview of New York City HIV care continuum dashboard, site-specific performance. CCD: care continuum dashboard; CD4: cluster of differentiation 4; NYC: New York City.

**Figure 2:**
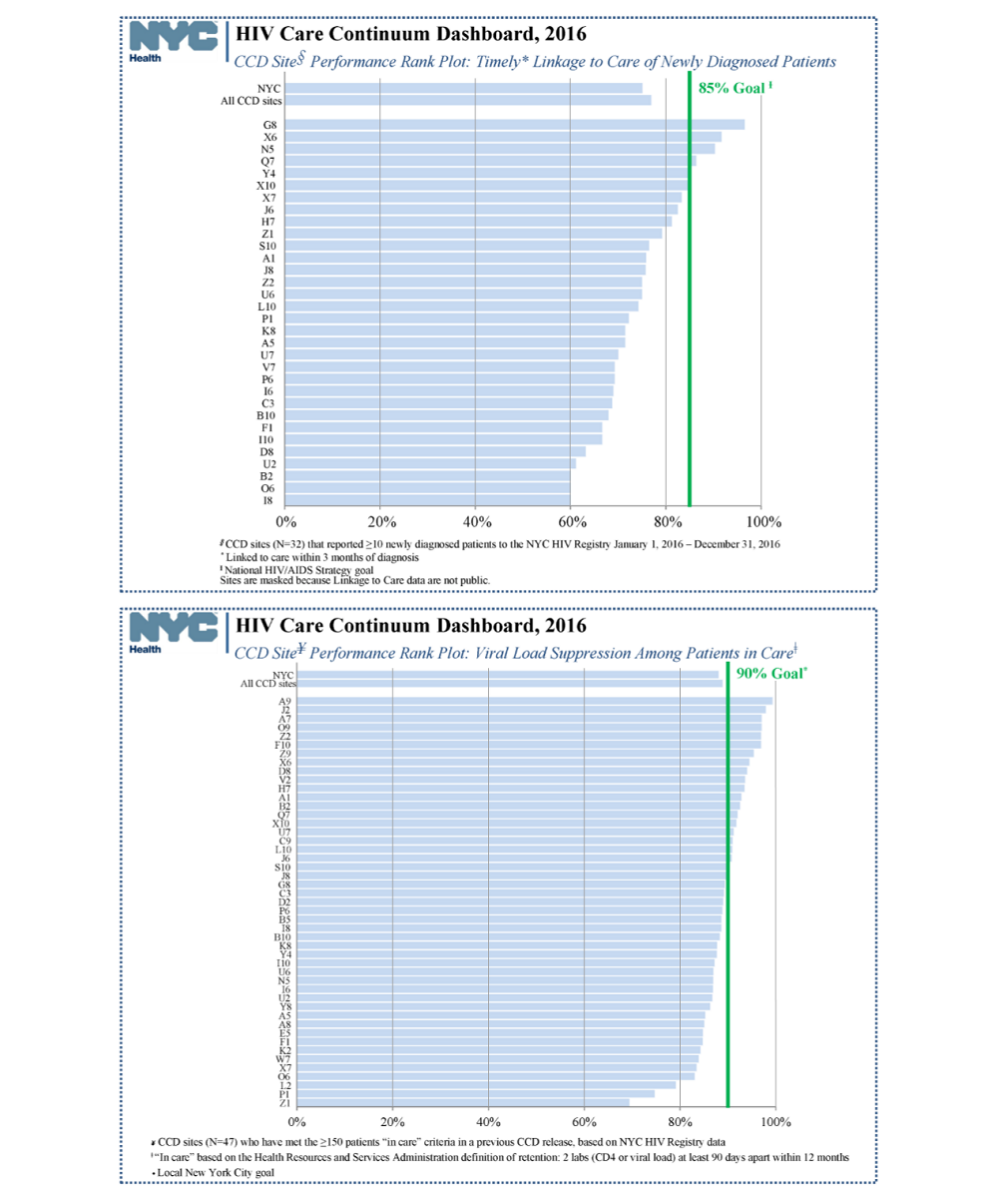
Overview of New York City HIV care continuum dashboard, linkage to care, and viral suppression rank plots. CCD: care continuum dashboard; CD4: cluster of differentiation 4.

The CCDs contain eligible facilities’ performance on LTC and VLS from the most recent analyzable 12-month period (Figure 1). The reports also include data from the site’s previous CCD so that facilities can evaluate their progress over time. Facilities also receive data on the proportion of their newly diagnosed patients that were linked at their facility versus at other New York City facilities. National and local targets are highlighted with goal lines for each indicator, enabling facilities to evaluate whether they meet the goals and make them aware of the current recommendations, as these targets can evolve with changes in national and local policy. In addition, facilities are provided with rank plots reflecting the LTC and VLS performance for all CCD sites during the same period, which allows comparison against peers’ performance (Figure 2). A frequently asked questions document is included in each release to assist facilities with interpreting the CCD. Finally, DOHMH has a dedicated email account to answer queries and provide assistance to sites.

#### Timely Linkage to Care

The timely LTC metric represents the proportion of newly diagnosed persons over 12 months at a given facility who linked to care within 3 months of their diagnosis. Linkage could have occurred either at the facility reporting the diagnosis or at any other facility in New York City. Timely LTC is considered to have occurred if any VL, CD4, or genotype test is ordered by the provider within 8 to 91 days of HIV diagnosis and reported to the DOHMH. HIV-related laboratory tests drawn within 7 days of a patient’s HIV diagnosis are likely to be part of the diagnostic work-up and therefore, do not represent LTC [18]. The timely LTC indicator is generated for facilities with 10 or more new diagnoses in the time period considered. The facility-based goal of 85% aligns with the LTC goal set forth in the 2010 and 2015 NHAS [10,11].

#### Viral Load Suppression

The VLS metric represents the proportion of persons whose most recent quantitative HIV VL was ≤200 copies/mL among all persons in care at the facility within the 12-month period of interest [19]. A patient is considered to be in continuous care at a facility if they have at least 2 HIV laboratory reports (CD4 or VL) 90 days apart or more ordered by this facility during the given 12-month time period. A patient who meets this definition at more than 1 New York City facility is only included in the CCD for the ordering facility of the patient’s last HIV laboratory test during the time period of interest. The VLS goal of 85% is based on examination of local New York City HIV surveillance data.

#### Viral Load Below Transmission Threshold

The “VL BTT” indicator represents the proportion of patients at a given facility whose most recent VL was <1,500 copies/mL among all persons in care (using the same definition of continuous care as for viral suppression) at that facility during the 12-month time period of interest. This indicator has both public health significance (as a measure of the proportion of patients with reduced potential to transmit HIV to partners) and clinical significance (as a measure of the proportion of patients who have low if not yet suppressed VL) [20,21]. In addition, this indicator serves as an adjustment for facilities that may care for populations with more barriers to achieving progress through the continuum of care. In exploratory analyses to develop the “VL BTT indicator” as an adjusted measure of viral suppression, no single demographic characteristic (eg, gender, race/ethnicity, age, borough of residence, and HIV transmission risk) of patients in care was useful for distinguishing those facilities that might have more complex patient populations and therefore more challenges to viral suppression. We therefore decided not to make specific adjustments to VL based on specific clinic populations but to create a generic indicator to capture the proportion of patients at each clinic who have low but not suppressed VL. VL<1500 copies/mL, which is lower than most VL set points in untreated individuals, implies an effort by a facility to treat patients with antiretroviral therapy (ART) and as such provides a crude estimate of the preventive impact of such treatment efforts in PLWH.

In more recent CCDs (those released in 2016 and 2017), the VLS target was moved to 90% from 85% in response to the substantial improvements over time in VLS among the CCD sites (see Figures 1 and 2). However, for easier comparison across years in this report, the VLS target was kept at 85%. A weighted average was used when assessing trends for a group of clinical sites to account for the relative size of the sites. In addition, low-performing facilities are defined as sites whose VLS or LTC is at least 10 percentage points below the 85% target.

## Results

### Program Implementation

**Figure 3:**
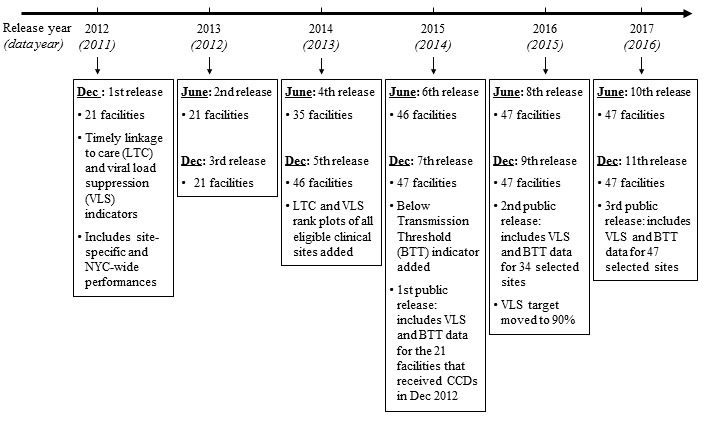
New York City (NYC) human immunodeficiency virus care continuum dashboards (CCDs) release timeline, 2012-2017. CCD releases are generated using data from a previous 12-month period to ensure completeness of the data (eg, the December 2012 release included data for January-December 2011; the June 2013 release included data for July 2011-June 2012, and so on). LTC: linkage to care; VLS: viral load suppression; BTT: below transmission threshold.

The first CCD release in December 2012 included data for 21 high-volume facilities (those with ≥1000 patients, and all New York City public hospitals). All site-affiliated clinics are included in a single facility CCD. In the December 2015 release, NYC DOHMH included 26 additional facilities (all with ≥150 patients in care), added the “BTT indicator,” and publicly released VLS data on DOHMH’s website for the 21 original CCD facilities. VLS data on all 47 CCD facilities have been publicly released since 2015 (Figure 3).

The 21 original sites whose data were released in December 2012 collectively covered 33.78% (1135/3360) of all newly HIV-diagnosed persons and 46.34% (28,405/61,298) of PLWH in care in New York City in 2011. In the December 2012 release, all 21 sites were eligible to receive LTC data for their newly diagnosed patients and VLS data for their patients established in care. Out of the 47 clinical sites that received CCDs in December 2017, 32 sites representing 41% of all new diagnoses were eligible to receive LTC data and all 47 were eligible to receive VLS data. These 47 sites collectively covered 44.23% of all newly diagnosed persons (1008/2279) and 69.59% (43,897/63,083) of PLWH in care in New York City in 2016.

### Impact on Outcomes

Table 1 shows the trends in VLS and LTC for the 21 original sites, which received all CCDs released during 2012 to 2017, reporting 2011 to 2016 data. The number of patients in care at the 21 sites remained relatively stable over the 4-year period. The weighted average VLS performance substantially increased from 73% in 2011 to 89% in 2016, with a sharp increase between 2012 and 2013 coinciding with the early roll-out of the recommendation of ART treatment for all. Improvements in VLS performance observed among the 5 lowest performing facilities were comparable with those among the 5 highest performing facilities, with a 13-point increase for both. The number of facilities meeting or exceeding the 85% target for VLS increased from 0 in 2011 to 16 in 2016. Moreover, there was a near-complete reduction in the number of low-performing sites performing at least 10 percentage points below target, from 13 in 2011 to 1 in 2016.

The number of newly diagnosed patients at the 21 original sites declined by about one-third between 2011 and 2016, from 1135 in 2011 to 720 in 2016. There was a modest increase in the weighted average performance on LTC (from 76%-77%) as well as a small increase in the number of facilities achieving the 85% national goal for linkage (from 2-4). The number of facilities performing at least 10 percentage points below target remained stable (from 11-12). In general, the declines in new HIV diagnoses, substantial increases in viral suppression, and more modest increases in LTC among the CCD sites are consistent with overall trends in HIV care outcomes in New York City during this period [22].

**Table 1:** 2011 to 2016 performance on viral load suppression and linkage to care for the 21 original care continuum dashboard sites.

Indicator		2011^a^	2012	2013	2014	2015	2016
**Viral load suppression (VLS)**							
	Patients in care, n	28,253	30,462	30,156	30,630	28,734	30,874
	VLS, weighted average (%)	73	73	84	86	88	89
	Sites above 85% VLS target, n	0	0	9	10	14	16
	Sites at least 10% below target, n	13	13	1	1	0	1
**Linkage to care (LTC)**							
	Diagnoses, n	1135	1024	991	914	769	720
	LTC, weighted average (%)	76	74	77	79	78	77
	Eligible sites (≥10 diagnoses), n	21	20	19	20	20	19
	Sites above 85% LTC target, n	2	1	2	4	4	4
	Sites at least 10% below target, n	11	10	7	8	6	12

^a^Recommendation for antiretroviral therapy for all people living with diagnosed HIV infection made in New York City in December 2011.

## Discussion

### Principal Findings

NYC DOHMH used the wealth of data in the HIV surveillance registry to create facility-level reports—the CCDs—that provide clinicians with valuable data for monitoring and improving the clinical management of PLWH in care in New York City. The CCD initiative fosters collaboration among HIV-care stakeholders, including providers, the Health Department, and patients. Ideally, this effort empowers providers to continuously raise their standards to ensure timely care of newly diagnosed patients, achieve undetectable VL for all PLWH, and ultimately prevent forward transmission of HIV in New York City.

Since the launch of the initiative, ongoing dialogue with some recipients has encouraged them to take ownership of the CCDs and use the reports to monitor their progress and address some clinical management issues rapidly. For example, 1 facility contacted the health department following the reporting of an unusually low VLS performance for their facility. This prompted an internal investigation by the facility, which revealed a lack of adherence to treatment among some of their patients and underscored the need for closer follow-up. The VLS performance of this facility has since substantially improved, although it remains below target. Another facility noticed important discrepancies between the LTC performance reported in their 2016 CCD and their internal estimates. On the basis of their feedback, NYC DOHMH investigated these discrepancies and found that some patients who had had their first care visit within 7 days after diagnosis failed to come back for an additional visit within 3 months. As a result of this observation, the facility became more aware of the importance of following up within 3 months with recently diagnosed patients for ongoing care engagement. We will monitor potential improvements in LTC at this site because of this investigation going forward. To date, such valuable feedback from sites has been received on a case-by-case basis. In the future, we are considering conducting a more systematic evaluation of the utility of the CCDs to recipient sites. In general, it is difficult to determine the full impact of the CCDs on patient outcomes at the sites because of the ecologic nature of the data and reports.

Ongoing discussions with providers have also helped to refine the methodology used in creating the CCDs to capture HIV care as accurately as possible. For example, VL and CD4 count were initially the only tests considered when assessing LTC. After consulting with providers, it was decided in 2016 to also include genotype tests as a marker of LTC. Going forward, advancements in biomedical therapies (eg, tenofovir alafenamide fumarate instead of tenofovir) might lead providers to draw laboratory tests less frequently, which could influence the validity of our current definition of retention in care. For example, it may be more effective to consider patients with 1 laboratory test or more in the year retained in care versus requiring 2 laboratory tests at least 3 months apart. Similarly, providers are being encouraged to link patients to care immediately after HIV diagnosis, and so changes to our 3-month linkage indicator in the near future are being considered.

The CCD initiative relies on the programmatic use of CD4, VL, diagnostic, and genotype tests that are reportable to DOHMH under New York State law. The use of these data for measuring and monitoring HIV care–related outcomes at the population level has been validated in previous studies [18-21,23,24]. New York City DOHMH has developed sophisticated procedures to ensure the quality, completeness, and timeliness of its HIV registry data. In particular, the NYC DOHMH registry integrates high-quality provider/facility-level data. Surveillance data can therefore be used to assess care received by a patient across all New York City facilities as opposed to the single facility-level view that is typically available to providers. High-quality data, an expanded purview for using surveillance data for public health action under New York State law, and local and national strategies that emphasize data-to-care approaches, have been cornerstones in the development of the CCDs.

### Limitations

The data used to generate the CCDs have inherent limitations. The New York City HIV surveillance registry contains laboratory tests ordered by New York City providers only. Thus, it fails to systematically capture patients who move or receive care outside of New York City. Therefore, patients who moved out of jurisdiction shortly after being diagnosed in New York City might appear as not timely linked to care (or not linked to care ever) despite successfully linking to care outside of New York City. Follow-up discussion with facilities focusing on patients in need of engagement in care often results in identification of such cases. Furthermore, clinic visits not associated with laboratory tests are not captured. This is an inherent limitation of using lab-based surveillance data to assess care engagement. However, previous validation work showed this discrepancy was relatively small [18]. In addition, to ensure completeness of data, CCDs are generated using data for a 12-month period plus a reporting lag. Moving forward, this lag time would ideally be shortened and enable closer to real-time reporting. Furthermore, facility-level VLS performance in the CCDs applies only to patients considered to be established in care at a provider based on the specific and relatively conservative definition of retention in care used for the CCDs; the definition fails to capture patients who are transitioning between providers as well as individuals who may be engaged in care but receiving less frequent VL monitoring. Despite these limitations, this definition allows us to capture and report on the care status of approximately two-thirds of New York City PLWH in care in the CCDs. Finally, by design, the CCDs do not include information on patients who are not in care or who have been lost to care. However, DOHMH maintains the HIV Care Status Reports system, which is a secure, Web-based system New York City providers can use to query information about lost-to-care patients against the surveillance registry for a check on patients’ current HIV care status in New York City.

### Conclusions and Implications

The CCDs are a novel approach to sharing aggregate-level surveillance data with providers who are responsible for the medical care of HIV patients. They enable the DOHMH and individual recipients to monitor progress toward national and local HIV care goals and monitor the quality of care delivery, detect areas for improvement, and inform the development of interventions (eg, training and technical assistance). Through the public release of VLS data, providers can identify peers with strong outcomes and engage in discussions regarding best practices. The CCDs also enable the identification of lower performing sites. To assist these sites, NYC DOHMH is developing a program in collaboration with New York State Department of Health to overcome possible barriers to the delivery of high-quality HIV care. Finally, public availability of CCD data promotes patient choice in where to seek HIV care. HIV surveillance data are a rich data source for monitoring outcomes such as engagement in care and viral suppression among PLWH in a jurisdiction and can be used effectively to monitor outcomes at the facility level. Similar initiatives can be adopted by other jurisdictions with mature surveillance systems and capacity and laws that support sharing surveillance data with providers.

## Abbreviations

ART: antiretroviral therapy
BTT: below transmission threshold
CCD: care continuum dashboard
CD4: cluster of differentiation 4
LTC: linkage to care
NHAS: National HIV/AIDS Strategy
NYC DOHMH: New York City Department of Health and Mental Hygiene
PLWH: persons living with HIV
VL: viral load
VLS: viral load suppression
